# Editorial: Advances in Ascochyta Research

**DOI:** 10.3389/fpls.2018.00022

**Published:** 2018-02-02

**Authors:** Diego Rubiales, Sara Fondevilla, Weidong Chen, Jennifer Davidson

**Affiliations:** ^1^Institute for Sustainable Agriculture, CSIC, Córdoba, Spain; ^2^USDA-ARS, Washington State University, Pullman, WA, United States; ^3^SARDI, Adelaide, SA, Australia

**Keywords:** Ascochyta blight, legumes, Lentil (*Lens culinaris*), Pea (*Pisum sativum*), Chickpea (*Cicer arietinum*), *Medicago truncatula*

Legume crops provide an excellent source of high quality plant protein and have a key role in arable crop rotations reducing the need for fertilizer application and acting as break-crops facilitating management of pests, diseases and weeds. However, these crops are themselves affected by a number of foliar and root diseases, with ascochyta blights being one of the most important groups of diseases worldwide. Ascochyta blights are incited by different pathogens in the various legume crops.

A number of control strategies have been developed including cultural practices and chemical control. However, only partial successes have been achieved since control methods can be uneconomical, hard to implement or result in incomplete protection. Nevertheless, the control methods available today represent major progress when compared to what was available one to two decades ago. Crops can be protected by cultural methods or by resistance, by selective fungicides, and by biocontrol agents, that did not exist before.

Infection of seed is one of the major survival mechanisms of *Ascochyta* spp. and an important means of transmission into previously uninfected areas. For some species this can also represent a major source of inoculum for the developing crop. Kumar and Banniza assessed the effect of seed infection with *A. pisi* on field pea in Canada. Although infected seeds may be an important way for the pathogen to survive in nature, their study concluded it cannot be regarded as a source of inoculum in the epidemiology of *A. pisi* under western Canadian growing conditions.

The use of resistant cultivars is widely acknowledged as the most economic and environmentally friendly control method. Breeding for ascochyta blight resistance has been a priority for breeding programs across the globe and consequently, a number of resistance sources have been identified and extensively exploited. However, ascochyta resistance breeding is not an easy task. The combination of genomic resources, effective molecular genetic tools and high resolution phenotyping tools will improve the efficiency of selection for ascochyta blight resistance and accelerate varietal development. Rodda et al. reviews current progress and future directions of molecular breeding for ascochyta blight resistance in lentil.

A detailed understanding of the genetic basis of ascochyta blight resistance is hence highly desirable, in order to obtain insight into the number and influence of resistance genes. Sudheesh et al. developed single nucleotide polymorphism (SNP)-based linkage maps from three recombinant inbred line (RIL) populations identifying totals of two and three quantitative trait loci (QTLs) explaining 52 and 69% of phenotypic variation for resistance in lentil. Evaluation of markers associated with ascochyta blight resistance across a diverse lentil germplasm panel revealed that the identity of alleles associated with one of the QTLs predicted the phenotypic responses with high levels of accuracy (~86%), and therefore have the potential to be widely adopted in lentil breeding programs.

Resistance breeding in legume crops has been slow due to the complex nature of resistance and the relatively low investment in genetics, genomics and biotechnology of legume crops, but also, mainly because of limited knowledge of the biology of the causal agents and pathogen variation. Davidson et al. investigated field reactions of lentil cultivars against *A. lentis* and the pathogenic variability of the *A. lentis* population in southern Australia on commonly grown cultivars, confirming the change in reaction on the foliage of the previously resistant cultivars. The impact of dominant cultivars in cropping systems and loss of effective disease resistance is discussed. Future studies are needed to determine if levels of aggressiveness among *A. lentis* isolates are increasing against a range of elite cultivars.

The recently reported changes in aggressiveness of *A. lentis* have led to decreased resistance within cultivars, reinforcing the utility of wild relatives as new sources of resistances. Dadu et al. reported novel resistance in wild lentil species *Lens orientalis*. This was consistently resistant against highly aggressive isolates recovered from diverse geographical lentil growing regions and host genotypes, suggesting stability and potential for future use of this resistance in lentil breeding.

A few major ascochyta blight R-genes have been characterized in different lentil genotypes. Sari et al. compared cellular and molecular defense responses to *A. lentis*. Histological examinations indicated that cell death triggered by the pathogen might be operative in some accessions, whereas limited colonization of epidermal cells might operate in others. Resistant accession differed also in timing and magnitude of SA and JA signaling pathway activation, corroborating the existence of diverse resistance mechanisms in lentil.

Large temporal and spatial variations have been detected within *Ascochyta* populations, and this can vary with the species and the region. Mehmood et al. showed that the Australian *A. rabiei* population has low genotypic diversity with only one mating type detected to date, potentially precluding substantial evolution through recombination. However, a large diversity in aggressiveness exists. In an effort to better understand the risk from selective adaptation to currently used resistance sources and chemical control strategies, the population was examined in detail concluding that the most common haplotype, ARH01, represents a significant risk to the Australian chickpea industry, being not only widely adapted to the diverse agro-geographical environments of the Australian chickpea growing regions, but also containing a disproportionately large number of aggressive isolates, indicating fitness to survive and ability to replicate on the best resistance sources in the Australian germplasm.

Temperature stresses might affect the resistance as well as pathogen aggressiveness. Kemal et al. showed that chilling temperature predisposed chickpea to *D. rabiei* infection. There were significant interactions of genotypes and isolates with temperature but this did not cause changes in the rank orders of the resistance of chickpea genotypes and aggressiveness of pathogen isolates.

Quinone outside inhibitor (QoI) fungicides (pyraclostrobin and azoxystrobin) have been the choice of farmers for managing ascochyta blight in pulses. However, Owati et al. detected and characterized resistance to these fungicides in *D. rabiei*. This indicates that where resistant isolates are located, fungicide failures may be observed in the field. *D. rabiei*-specific polymerase chain reaction primer sets and hydrolysis probes were developed to efficiently discriminate QoI-resistant from QoI-sensitive isolates.

The genetic resistance to ascochyta blight in chickpea is complex and governed by multiple QTLs. The molecular mechanism of quantitative disease resistance to ascochyta blight and the genes underlying these QTLs are still unknown. Most often disease resistance is determined by resistance R-genes, the most predominant of which contain nucleotide binding site and leucine rich repeat (NBS-LRR) domains. Sagi et al. performed a genetic analysis of NBS-LRR gene family in chickpea and their expression profiles in response to ascochyta blight infection. Thirty of the NBS-LRR genes co-localized with nine of the previously reported ascochyta blight QTLs. Of these, 27 showed differential expression in response to ascochyta blight infection.

Li et al. sequenced a collection of resistant chickpea genotypes, and identified more than 800,000 SNPs. Population structure analysis revealed relatively narrow genetic diversity amongst recently released Australian varieties and two groups of varieties separated by the level of ascochyta blight resistance. A 100 kb region (AB4.1) on chromosome 4 was significantly associated with ascochyta blight resistance collocating to a large QTL. This region was validated by GWAS in an additional collection of 132 advanced breeding lines. This study demonstrates the power of combining whole genome re-sequencing data with relatively simple traits to rapidly develop “functional makers” for marker-assisted selection and genomic selection.

Verma et al. performed a genome-wide identification and analysis of transcription factors (TFs) in *A. rabiei*, taking advantage of *A. rabiei* genome sequence. The *A. rabiei* secretome was predicted to be mainly regulated by Myb TFs. Expression profile of TFs varied with pathotype of *A. rabiei* and the cultivar of chickpea. The analyses would provide the basis for further studies to dissect the molecular mechanisms of *A. rabiei* pathogenesis.

The species *Didymella pinodes* is the principal causal agent of ascochyta blight, one of the most important fungal diseases of field pea worldwide. Understanding its host specificity has crucial implications in epidemiology and management. Barilli et al. delineated the host range of *D. pinodes* among legume crops and wild relatives, and compared it with that of other close species. *D. pinodes* was highly virulent on field pea accessions, although differences in virulence were observed among isolates. *D. pinodes* host range is larger than that of *D. fabae, D. lentil*, and *D. rabiei*. This has relevant implications in epidemiology and control as these species might act as alternative hosts for *D. pinodes*.

Suzuki et al. examined the histology and ultrastructure of early infection events and fungal development in the pathosystem *Medicago truncatula*/*D. pinodes*. Successful penetration and subsequent growth of infection hyphae were considerably restricted in the resistant ecotype. The oxidative burst reaction leading to the generation of reactive oxygen species is associated with a local host defense response in the resistant ecotype, since no clear H_2_O_2_ accumulation was detectable in the susceptible ecotype.

QTL mapping studies in several field pea crosses have resulted in identification of genomic regions associated with ascochyta blight resistance. However, these QTLs cover large regions which may not be effective for marker-assisted selection. Jha et al. fine mapped two of these QTLs using a high density SNP-based genetic linkage map and identified markers in heterogeneous inbred family populations. Resistance to lodging was also associated with these two QTLs. The identified SNP markers will be useful in marker assisted selection for development of field pea cultivars with improved ascochyta blight resistance.

## Author contributions

DR, SF, WC, and JD were guest editors of the RT. The four of them contributed to this editorial.

### Conflict of interest statement

The authors declare that the research was conducted in the absence of any commercial or financial relationships that could be construed as a potential conflict of interest.

